# Bibliographical

**Published:** 1880-01

**Authors:** 


### EDITORIAL.

### BIBLIOGRAPHICAL.

### THE ADVANTAGES AND ACCIDENTS OF ARTIFICIAL ANÆSTHESIA.

*A Manual of Anæsthetic Agents and their Employment in the treatment of Disease.*

BY LAWRENCE TURNBULL, M. D., PHILADELPHIA.

*Second Edition revised and enlarged with Twenty New Illustrations-*

This work is just issued from the house of Lindsey & Blakeston. It is but a short time since the first edition was issued, but so highly was it appreciated, that it was exhausted within about one year, and a second was demanded.

Anæsthesia is a subject of great interest and importance to physicians and dentists, and everything that will aid them in better understanding the subject, and better and more safely using the agents, is sought with great avidity. More or less of danger attaches to nearly or quite all these agents, and the greatest security from this is an important matter.

In the preface, the author says: "In this second edition there will be found many more practical suggestions, as to the employment of anæsthetics that are safe, and the rules for their adoption, or reasons for the rejection of some of them in different cases, grouped and made convenient, so that the student can manage them and be fully prepared for any emergency.

The merits of the various anæsthetic agents, are fully discussed, and the classes of cases for which each is adapted. The methods of guarding against danger, are fully presented and discussed, also the mode of procedure in an emergency.

These are all vital points, and should receive the most scrutinizing attention and study of every one who administers these agents.

This work we regard as the best aid in the study of this subject, now available. It has been prepared by one who has had large experience, and in this work has demonstrated his ability to present the subject in a very compact and practical form; and it presents the subject up to the present hour. It is obtainable of Robt. Clarke & Co. of this city, and of book-sellers generally.

### PRACTICAL INFORMATION ABOUT THE TEETH.

A. HOLBROOK, D. D. S., MILWAUKEE, WIS.

This little work supplies, in a complete and popular form, a mass of condensed information, which, if read and digested by the laity, would greatly aid the practioner in his endeavors to preserve the natural teeth. In a succinct manner, the author sketches the anatomy of the teeth, their position, general character, structure, etc. The pre-disposing and acting causes of decay are treated of in an intelligent way, covering the salient points, and giving a good idea of the difficulties the dentist is expected to overcome. The subject of food receives a large share of attention, impressing upon the reader, the importance of proper diet. The constitutional and local treatment of diseases of the mouth and teeth, are both concisely described. The illustrations, tables, and formulæ throughout the work, very materially assist in making it intelligible.

### AMERICAN HEALTH PRIMERS.
### VOL. VI.—WINTER AND ITS DANGERS.

BY HAMILTON OSGOOD, M. D., BOSTON.

Lindsay and Blakiston have just issued the sixth volume of this valuable series. The writer of this little work has, indeed, given us a Christmas present of great practical value, and if we study it and apply the knowledge set forth, we could not better repay him for his efforts in our behalf. Coming at this season of the year, when all, at some time or another, are sufferers to a greater or lesser extent from some cause we very likely did not perceive, this "Winter's Tale" of modern hygienic medicine, fills a niche, long vacant in the family bookcase. Errors in dress; the ignorant use of the bath; inattention to pulmonary food; danger from overheated air; neglect of sunshine; sedentary habits; school life in winter, and winter amusements, all furnish the subject-matter, which, in the hands of Dr. Osgood, becomes not only instructive and practical, but entertaining, as well.

To be had of Rob't Clarke & Co.

### STUDENTS' POCKET MEDICAL LEXICON,

*With an Appendix containing a list of Poisons and their Antidotes, Abbreviations used in Perscriptions, and a Metric Scale of Doses.*

BY ELIAS LONGLEY.

This condensed and comprehensive little work just issued by Lindsey and Blakiston, is a valuable one to the medical or dental students. It gives the correct pronunciation and definition of all the words and terms in general use in medicine, and the collateral sciences. The pronunciation is plainly represented in the American Phonetic Alphabet.

This is the most complete work of the kind extant, so far I am aware. As a book of daily reference for the pronunciation and definition, it will often take the place of the more pretentious volumes, at least render their use far less frequent. The size, which renders it a constant companion to him who desires it renders it valuable to the student of medicine in any or all of its branches.

For sale by Robt. Clarke & Co. Price $1.00.

